# A Hydrazine Coupled Cycling Assay Validates the Decrease in Redox Ratio under Starvation in *Drosophila*

**DOI:** 10.1371/journal.pone.0047584

**Published:** 2012-10-17

**Authors:** Chen-Tseh Zhu, David M. Rand

**Affiliations:** Department of Ecology and Evolution Biology, Brown University, Providence, Rhode Island, United States of America; Florida State University, United States of America

## Abstract

A commonly used enzymatic recycling assay for pyridine nucleotides has been adapted to directly measure the NAD^+^/NADH redox ratio in *Drosophila melanogaster*. This method is also suitable for quantification of NADP^+^ and NADPH. The addition of a coupling reaction removing acetaldehyde produced from the alcohol dehydrogenase (ADH) reaction was shown to improve the linearity of NAD(H) assay. The advantages of this assay method are that it allows the determination of both NAD^+^ and NADH simultaneously while keeping enzymatic degradation of pyridine nucleotides minimal and also achieving better sensitivity. This method was used to determine the redox ratio of *D. melanogaster* and validated substantial decrease of redox ratio during starvation.

## Introduction

The levels of pyridine nucleotides and their redox ratios, NAD^+^/NADH and NADP^+^/NADPH, are important biological signatures of metabolic status and are believed to be useful biomarkers of aging, disease and transcription regulation [1], [2], [3], [4], [5], [6], [7], [8], [9], [10], [11], [12], [13], [14]. Many different approaches have been proposed to measure these ratios, either indirectly by quantifying the concentration of substrates and products in NAD^+^ dependent dehydrogenase reactions, or more directly by HPLC, NMR or MS [15], [16], [17]. *In vivo* quantification methods have been developed as well (reviewed in [18]). Among these, the enzymatic cycling assay is a convenient, fast and reliable approach to estimate the redox ratio [19], [20], [21], [22], [23]. It does not require sophisticated equipment as it can be performed easily in 96-well microplates, and quantified using an absorbance or fluorescence plate reader.

**Figure 1:**
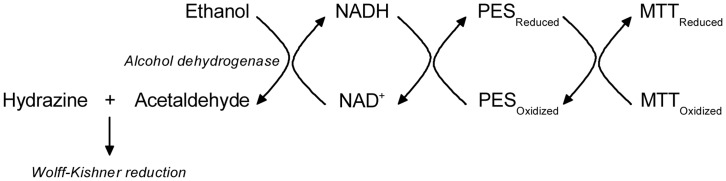
A representative scheme of a cycling assay for pyridine nucleotides. In this case, the oxidation of ethanol to acetaldehyde catalyzed by ADH is used to assay NADx. The redox indicator is a MTT/PES coupled reaction. Acetaldehyde is removed by reacting with hydrazine in a Wolff-Kishner reduction (see detail in text).

The principle underlying an enzymatic cycling assay is illustrated below (Fig. 1). This principal method was invented by Lowry *et al*. and subsequently modified and improved [19], [20], [21], [22], [23]. In the presence of an NAD^+^ dependent dehydrogenase (e.g. ADH, alcohol dehydrogenase, E.C. 1.1.1.1), NAD^+^ is reduced to NADH. Once formed, reduced pyridine nucleotides donate electrons to MTT (3-(4,5-Dimethylthiazol-2-yl)-2,5-diphenyltetrazolium bromide) in a PES (phenazine ethosulfate) coupled reaction, resulting in a purple formazan product that can be quantitatively measured at a wavelength of 570 nm. In this system, pyridine nucleotides are recycled between oxidized and reduced form, eventually passing the electron from ethanol to a redox indicator dye, hence the term ‘cycling assay’, or more precisely, reactant recycling assay. When only the concentration of pyridine nucleotides is limited, the overall rate is proportional to the total amount of NAD^+^ and NADH (NADx hereafter) in the reaction.

Since the assay will not distinguish between reduced and oxidized pyridine nucleotides, to measure NAD^+^/NADH redox ratio one has to use another method to distinguish the two states. From the studies of stability of pyridine nucleotides, it has long been known that the reduced pyridine nucleotides are rapidly degraded in low pH while stable in alkali [24], [25]. The oxidized form, on the other hand, is unstable in alkali but stable in acid. Increasing temperature or PO_4_^3−^ concentration increases the degradation rate [24]. Utilizing these instability differences of reduced and oxidized forms, two approaches were proposed to distinguish them. In one approach, sample is extracted and the extraction is aliquotted into 2 parts. One aliquot is treated at 65°C to degrade NAD^+^ and subsequently measured for NADH only; meanwhile the other aliquot, which is not heat treated, can be assayed for the sum of NADH and NAD^+^ [15], [23]. In the other approach, the same sample is divided and extracted in two different solutions: the alkali extraction for NADH and the acid extraction for NAD^+^. Both extractions will then be adjusted to neutral pH prior to performing the recycling assay to determine the concentration of pyridine nucleotides [19], [21], [26].

In this study, we developed a method to extract total NADx from whole fruit flies while minimizing enzymatic degradation during sample preparation. We also modified the existing extraction procedure so that both oxidized and reduced state can be measured from the same homogenate and NAD^+^/NADH ratio can be directly calculated, saving the effort of introducing an external control (e.g. protein concentration or weight) if NAD^+^ and NADH are extracted separately. We found this approach to be also suitable for assaying NADPH and NADP^+^ (NADPx hereafter) with small changes in the protocol. For the NADx assay that relies on ADH, we found a simple way to greatly improve the reaction linearity and assay sensitivity for this enzyme over a wide range. Finally, we applied this assay to *Drosophila melanogaster* subjected to a period of starvation and verified the expected changes in redox ratios that accompany decreases in energy stores.

The list of abbreviations used in this reported is summarized as Table S1.

## Materials and Methods

### Reagents

The homogenization buffer consisted of the following reagents: 10 mM nicotinamide, 10 mM Tris-Cl, 0.05% (w/v) Triton X-100, pH 7.4 adjusted using HCl. The presence of nicotinamide is to reduce enzymatic degradation by enzymes such as by ADP-ribosyltransferases. The reaction mixture for the NADx assay contained: 0.1 M BICINE (N,N-bis(2-hydroxyethyl)glycine), 0.6 M ethanol, 50 mM EDTA, 2 mM PES and 0.5 mM MTT. PES and MTT were prepared as 25× stock solutions of 50 mM and 12.5 mM in water respectively. The reaction mixture for the NADPx assay was the same as NAD assay mixture except for the substrate: 50 mM glucose 6-phosphate (G6P) instead of ethanol. For fluorescence assay, PES and MTT were substituted by PMS (phenazine methosulfate) and resazurin at final concentrations of 0.5 mM and 50 µM respectively prepared as 100× stock water solution. Among all the reagents, the PES water solution is highly unstable and needs to be made fresh. MTT, PMS and resazurin are more stable than PES and stock solutions can be aliquotted into single use vials and stored in −20°C for at least a week. All dyes were kept away from direct light before being added into reaction mix.

Phenol: chloroform: isoamyl alcohol (25∶24:1 v/v) was saturated with 100 mM Tris-HCl buffer (pH 7.4–8.0) as phenol is a weak acid. Chloroform was saturated with homogenization buffer described above.

10 mM NAD^+^ or NADP^+^ standard solutions were prepared fresh in homogenization buffer. NAD^+^ or NADP^+^, rather than their reduced forms were the preferred standards due to longer shelf life in water solution. Once made, they can be kept on ice away from light for up to at least 5 hours without degradation, but not overnight.

For NAD assay, final concentration for ADH was 0.2 mg protein/ml. 25× stock solution (5 mg/ml) was prepared fresh from lyophilized enzyme powder (337 unit/mg protein) for each experiment. For NADPx assay, the NADP^+^ dependent glucose 6-phosphate dehydrogenase (G6PDH) was employed as the cycling enzyme. A final concentration of 0.2 unit/ml G6PDH was used.

A list of all the required reagents, with their supplier catalog numbers, is provided as Table S2.

### Extraction of Pyridine Nucleotide from Whole *Drosophila*

Fifteen male flies (or 10 females, approximately 10 mg wet weight) were anesthetized by CO_2_ and homogenized immediately in 250 µl of homogenization buffer. The homogenate was centrifuged at 12000×g for 1 min. Supernatant was collected and a small fraction was kept aside to determine soluble protein concentration. The remaining supernatant was treated with equal volume of phenol: chloroform: isoamyl alcohol (25∶24:1, v/v), mixed vigorously and centrifuged at 12000×g for 5 min. The aqueous phase was collected and subjected to another round of extraction using an equal volume of chloroform and centrifuged at 12000×g for 5 min. The resulting aqueous phase contains pyridine nucleotide.

Two aliqouts of 18 µl of the pyridine nucleotide extraction were removed. One aliquot was mixed with 2 µl of 0.1 M HCl and the other with 2 µl of NaOH so that the final [H^+^] or [OH^-^] were 0.01 M. They were then both heated on a 65°C heat block for 30 min to degrade the reduced or the oxidized pyridine nucleotide respectively and were immediately chilled on ice. Finally, 2 µl of the opposite reagent (NaOH or HCl) wes added to neutralize pH.

Homogenization, extraction and centrifuging steps were performed at 4°C.

### Plate Reader Settings

When using PES and MTT as detection dyes, the plate reader should be set to measure absorbance at 570 nm. When using PMS and resorufin, a plate reader capable of taking fluorescence reading was configured such that the excitation wavelength was 540 nm and emission wavelength was 586 nm. Kinetic curves are recorded for the first 5 to 10 minutes. In theory, one should be taking the initial rate of reaction. We however took data points from 60 s to 300 s and calculated average reaction velocity (*V_mean_)* as often the enzyme requires a short period to become most active. We used SpectraMax Plus and SpectraMax M5 96-well plate readers from Molecular Devices (Sunnyvale, California, U.S.) for absorbance and fluorescence assays respectively. The reading interval is largely instrument dependent but is also affected by the number of samples. We always choose the shortest possible interval (no longer than 10 s). Readings were all performed at temperature about 37°C.

### Plate Assay Protocol

The NAD^+^ standard curve was made by 2-fold serial dilution using homogenization buffer starting from 100 µM. 5 µl of NAD^+^ standard or sample were added to each well containing 120 µl of reaction mixture without PES and MTT, which were added to start the reaction right before the plate was read. After adding the dyes, dehydrogenase was added into the reaction mixture and reading started. Adding PES only shortly before reading was proposed by Umemura and Kimura [23] as an approach to minimize background noise. The blank control assay contained reaction mixture and 5 µl of homogenization buffer only.

A standard curve was generated by plotting the concentrations of standards against *V_mean_* and the concentrations of unknown samples were calculated from interpolation.

### Triacylglyceride, Glycogen, Glucose and Soluble Protein Quantification

Homogenates not treated with phenol-chloroform were used for these assays. Triacylglyceride was assayed using glycerol phosphate oxidase (GPO) based method (Infinity™ Triglycerides reagent, Thermo Scientific). Glycogen and glucose were assayed using glucose oxidase based method (Infinity™ Glucose reagent, Thermo Scientific). Glycogen was converted to glucose using amyloglucosidase (Roche Diagnostics) and was subsequently assayed. Soluble protein concentration was determined using BCA Protein Assay Reagent (Thermo Scientific Pierce).

### Statistical Methods

Methods for estimating and testing the difference of redox ratio in different treatment group are included as Materials and Methods S1 unless otherwise described in the text.

## Results

### Typical Result for NADPx and NADx Assay, and the Optimization of ADH Based NADx Assay

**Figure 2:**
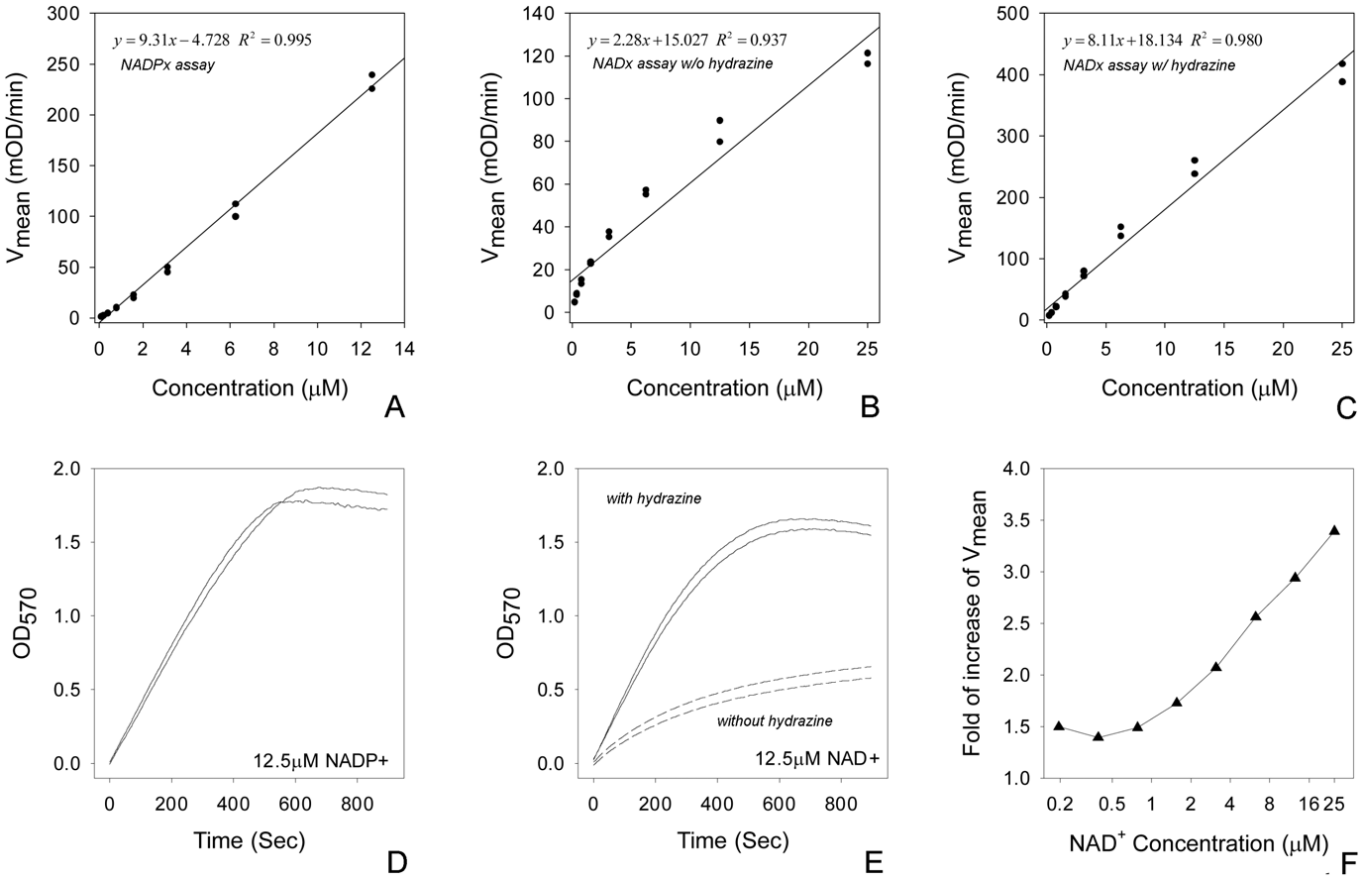
Comparison of reaction kinetics of NADPx, NADx assay with and without hydrazine. A: Standard curve of NADPx assay. B: Standard curve of NADx assay without hydrazine. C: Standard curve of NADx assay with hydrazine. D: reaction kinetic of NADPx assay. E: NADx assay kinetic, showing additional hydrazine increases *V_mean_*. F: the increase of *V_mean_* by addition of hydrazine is dependent on the concentration of NAD^+^. All assays were performed in duplicates and hydrazine concentration is 0.02%.

Typical standard curve and reaction kinetics for NADPx using PES/MTT assay are shown in Figure 2a and 2d. As more MTT is being reduced, the color of the assay mixture changes from yellow to green. However, over time, the reduced MTT forms precipitates which leads to a gradual decline of absorbance once it reached its peak OD_570_ of about 1.7 (Fig. 2d).

A typical standard curve for NADx assay is shown in Figure 2b. Compared to the NADPx assay, the NADx assay is less linear and has a much smaller slope (compare y-axes of Fig. 2a and Fig. 2b). The reaction velocity decreases over time resulting in blended kinetics curves indicating either the enzyme activity or ethanol is limiting (Fig. 2e, “Without hydrazine”). We found increasing the final concentration of enzyme, even up to 200 µg/ml, does not remedy the problem. The average reaction rate seems to be proportional to the concentration of ethanol (data not shown). As the oxidation of ethanol to acetaldehyde has a positive ΔG^’0^ of 47.2 kJ/mol, a relatively high concentration of ethanol is used in the reaction mixture in order to push the reaction forward. High ethanol concentration alone, although lowering the free energy change, does not improve the assay dramatically, as acetaldehyde is not removed and may eventually inhibit the reaction by blocking the enzyme catalytic site.

**Figure 3:**
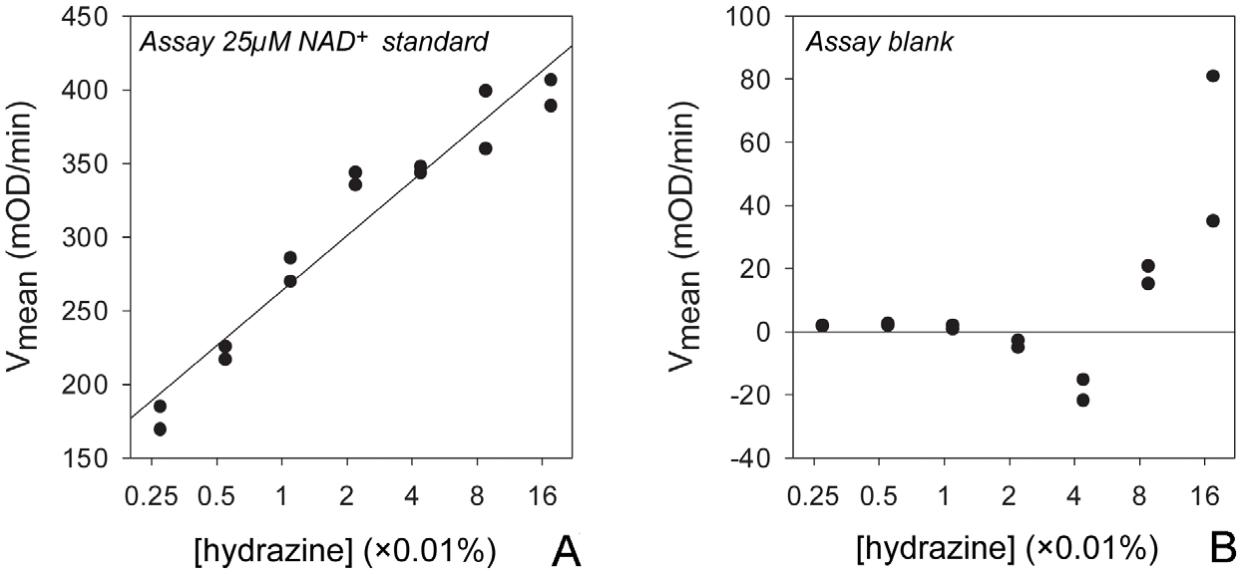
The optimal concentration of hydrazine is around 0.02%. A: In a reaction containing 25 µM NAD^+^, *V_mean_* increases with hydrazine concentration log-linearly up to 0.02%. B: The rate of absorbance increase in no NAD^+^ blank control is affected by hydrazine concentration higher than 0.02%.

We chose to couple the ethanol to acetaldehyde reaction with a reaction utilizing acetaldehyde to solve this problem. One possibility is to couple it to the acetaldehyde oxidation catalyzed by acetaldehyde dehydrogenase (ALDH), which has a ΔG^’0^ of −215.12 kJ/mol. Another possibility is to couple it to Wolff–Kishner reduction, where acetaldehyde reacts with hydrazine eventually forming N_2_ and ethane. We found adding a final concentration of 0.02% hydrazine (supplied with 35% w/w hydrazine water solution, Sigma-Aldrich #309400) into reaction mixture largely solved the problem (Fig. 3a). *V_mean_* increases linearly with log[hydrazine] (Fig. 3a) but at concentrations higher than 0.02% the OD increase of the blank becomes large (Fig. 3b). Hydrazine increases *V_mean_* of ADH reaction in an [NADx] dependent fashion (Fig. 2f) as well. In all, hydrazine maintains reaction velocity and improves the linearity of standard curve (Fig. 1b, 2c and 2e).

It is possible to use less ADH in the NADx assay when it is coupled with Wolff–Kishner reduction. Assaying 25 µM NAD^+^ with 80, 120, 160 and 200 µg/ml ADH, we found ADH concentration (mg/ml) and *V_mean_* to have a weak and only marginally significant relationship (*V_mean_* = 379+0.558[ADH], *p* = 0.047, n = 8).

### 65°C Incubation for 30 Minutes is not Sufficient to Fully Degrade NAD^+^

Thirty minutes of 65°C incubation was shown to degrade NAD^+^ while keeping NADH intact [24], as NAD^+^ has a higher temperature dependent degradation rate than NADH at pH around 7.0 [24]. We find such a treatment is not sufficient for *Drosophila* homogenate and additional 0.01 M OH^−^ is required in order to fully decompose NAD^+^.

NAD^+^ standard solutions of 25, 12.5, 6.25 and 3.125 µM were diluted 5 fold with either homogenization buffer or fly homogenate. Subjected to different heat and pH treatment, these diluted NAD^+^ standards were subsequently assayed by enzyme cycling. The slopes between the rate of absorbance increase (*V_mean_*) and the concentration of NAD^+^ standard are summarized in Table 1. When diluted in *Drosophila* homogenate, 30 min of 65°C incubation did not fully destroy all NAD^+^ as implied by a significant positive slope between *V_mean_* and the amount of NAD^+^ added by standard solution. Only with additional 0.01 M OH^-^ present in *Drosophila* homogenate did this slope become not different from zero indicating all NAD^+^ was degraded. It appears fly homogenate is able to block the heat-related degradation of NAD^+^. The results are summarized in Table 1.

**Table 1:** Slopes between the rate of absorbance increase (*V_mean_*) and the concentration of NAD^+^ (10^−4^·OD·min^−1^·µM^−1^) in either homogenate or homogenization buffer.

NAD^+^ diluted in:	Treatment		Slope	*r^2^*
fly homogenate	30 min, 65°C		2.820	0.793, **
	30 min, 65°C,	0.01 M OH^−^	0.027	0.009, *N.S.*
	30 min, 65°C,	0.01 M H^+^	15.875	0.965, ***
homogenizationbuffer	30 min, 65°C,	0.01 M OH^−^	0.104	0.244, *N.S.*
	30 min, 65°C,	0.01 M H^+^	14.657	0.997, ***

*N.S.*: non-significant; **, *p*<0.01; ***, *p*<0.001. Fly homogenate is generated by homogenizing 15 male *D. melanogaster* adults in 250 ul homogenization buffer. Particles were removed by 5 min 16000×g centrifugation.

However, we found fly homogenate does not appear to block the degradation of NADH, which is stable in 65°C with 0.01 M OH^−^ and quickly degraded in the same temperature in 0.01 M H^+^ as found by Lowry *et al*. [24] (Data not shown).

### Phenol-chloroform Extraction Protects Pyridine Nucleotide from Enzymatic Degradation during Preparations and Dissociates Protein-bound NADH

**Figure 4:**
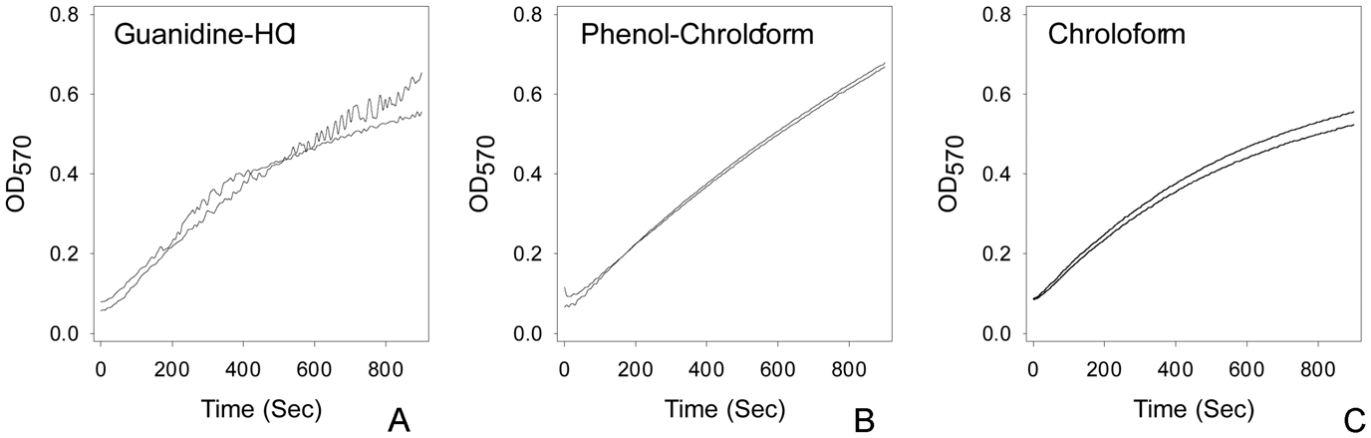
Comparison of three different extraction methods. Three sets of 15 *D. melanogaster* adult males were subjected to different treatments. Homogenized in buffer with additional 6 M guanidine-HCl or: homogenized and treated with equal volume phenol-chloroform or chloroform only. The samples were then assayed for NADx in duplicates.

It has long been known that most of the NAD^+^ exists in free solution form while most of the NADH is protein bound [27]. In order to accurately measure the total amount of NADH, the extraction method should be able to denature protein and release NADH. Denaturing protein will also reduce enzymatic degradation of pyridine nucleotides. We compared three extraction methods: phenol chloroform extraction [28], chloroform-only extraction and 6 M guanidine-HCl. Guanidine-HCl extraction is a strong denaturing reagent which is capable of breaking protein secondary structure and has been used in assaying other metabolites [29], [30], [31]. We found guanidine extraction interfered with ADH activity (Fig. 4a) and is not suitable for this enzymatic recycling assay. For reasons unknown, phenol-chloroform treated samples also give a more linear kinetic curve than that of chloroform-only treated samples (Fig. 4a and 4b).

We compared the redox ratio (NAD^+^/NADH) obtained from the same fly tissue homogenate: untreated control, chloroform-only treated and phenol-chloroform treated (see Table 2). Phenol-chloroform treatment gives the lowest value, suggesting NADH recovery in this reaction is the highest among all three. Also noticeable is that the amount of pyridine nucleotides detected following phenol-chloroform extraction is the highest among three treatments.

**Table 2:** Phenol-Chloroform extraction is efficient for extracting NADH.

	(µmol/g protein)					
ExtractionMethod	NAD^+^		NADH		Redox Ratio	
Control	5.933	**Compared**	1.084	**Compared**	5.114	**Compared**
	±0.125	**to Control**	±0.087	**to Control**	±0.419	**to Control**
Chloroform	4.959	***	0.915	*N.S.*	4.871	*N.S.*
	±0.095	*p*<0.001	±0.090	*p* = 0.192	±0.516	*p* = 0.717
Phenol-Chloroform	7.159	***	1.869	***	3.820	**
	±0.119	*p*<0.001	±0.036	*p*<0.001	±0.073	*p* = 0.002

Six samples of 15 male *D. melanogaster* adults were homogenized in 250 ul homogenization buffer. Following a 5 min 16000×g centrifugation, the supernatant was then divided into 3 parts. One part was kept as control. The other two parts were treated with equal volume of chloroform and phenol-chloroform respectively. Three parts were then assayed for NAD^+^ and NADH in duplicates. The concentration of NAD^+^ and NADH, with S.E.M, are standardized by the concentration of soluble protein measured from the control group. The difference in NAD^+^ and NADH concentration is tested using two sample *t*-test. See supplementary material for the method of testing redox ratio difference between groups.

### Redox Ratio Decreases when Flies are Exposed to Short Starvation

The redox ratio (NAD^+^/NADH) decreases during starvation, which was first shown by Williamson *et al*. [27]. During starvation, metabolism switches to storage energy utilization, including β–oxidation of fatty acid, which leads to the generation of ketone bodies by excess level of Acetyl-CoA. Theses changes result in a reduction of the redox ratio, which was first shown by measuring, rather indirectly, the ratio of reactant and product of dehydrogenase reaction [27]. A number of dehydrogenase reactions were known to be at or near equilibrium such as Malate dehydrogenase (EC 1.1.1.37, Mdh). The ratio between free concentrations, [NAD^+^]/[NADH], can be calculated when [malate]/[oxaloacetate] and the equilibrium constant are known [27]. Here using the enzyme recycling assay directly measuring total amount of pyridine nucleotides, we show that the ratio NAD^+^/NADH decreased under starvation.

Fifteen newly eclosed male flies of the *w^1118^* strains were collected and aged to day 10 on 10% yeast, 10% sugar and 2% (w/v) agar food vials. Vials were kept in 25°C, 60% relative humidity incubator with a 12∶12 hours light cycle (light-on at 8∶00 a.m.). At 12∶00 a.m. on the 10th day, they were transferred to vials containing 2% (w/v) agar, which cannot be utilized as a food source by *Drosophila*, and assayed for NAD^+^, NADH, NADP^+^ and NADPH after 10 hours. Starting starvation treatment at Zeitgeber time +16 hours is to minimize food intake variation among animals, as it was clearly demonstrated by Xu *et al*. that feeding activity is at its minimal at this time [32].The control group was kept on the aforementioned yeast-sugar-agar food for the same amount of time.

As summarized in table 3, we found that NAD^+^/NADH redox ratio of well-fed *Drosophila* is around 8, and with 10 hours of starvation the redox ratio decreases to about 4 which is highly significant.

**Table 3:** The level of NADx and NADPx of fed and starved flies.

	(µmol/g protein)				Redox Ratio			
Treatment	NAD^+^	NADH	NADP^+^	NADPH	NAD^+^/NADH		NADP^+^/NADPH	
Fed	3.994	0.493	0.083	0.336	8.093		0.244	
	±0.203	±0.013	±0.006	±0.010	±0.270	*p*<	±0.016	*p*<
Starved	3.878	0.868	0.042	0.324	4.468	0.001	0.127	0.001
	±0.105	±0.020	±0.001	±0.010	±0.095		±0.005	

The concentration of pyridine nucleotides with S.E.M, are standardized by the concentration of soluble protein measured prior to phenol-chloroform extraction. Statistical analysis follows the same linear regression model as in Table 2. n = 8 and measured in duplicates.

The ratio of total NADP^+^/NADPH was found to be around 0.2. It also decreases after 10 hours of starvation (Table 3).

**Figure 5:**
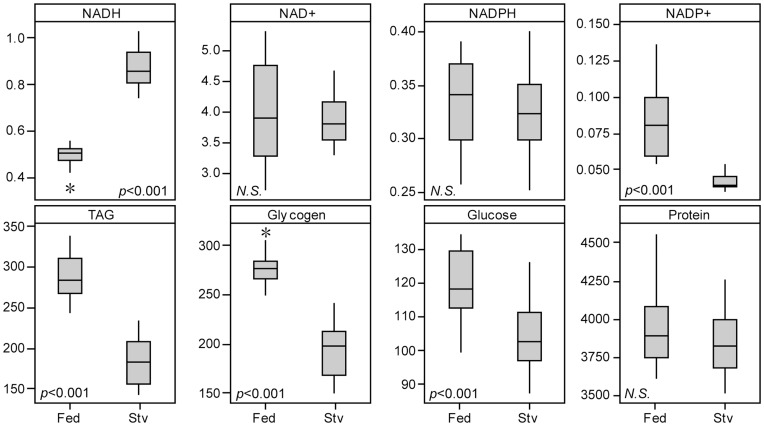
Changes in the concentrations of protein and metabolites following starvation. Comparisons are between flies held on food (‘Fed’) and on 2% agar (starved, ‘Stv’ on x-axes). Unit: µmol/g protein For NADH, NAD^+^, NADPH and NADP^+^. mg/g protein for TAG, glycogen and glucose. µg/ml for protein. n = 8 and measured in duplicates. The between group differences are tested by 2 sample *t*-test.

We verified the animals were truly in a starvation state by measuring the level of triacylglyceride, glycogen and glucose and detected their levels in the starved group were significantly lower (Fig. 5). We found the concentration of protein is not significantly affected by starvation (2 sample *t*-test, *p = *0.342). On the other hand, the mean survival time during starvation for strain *w^1118^* is about 20 hours (data not shown). By choosing a relatively short starvation duration, this experiment captures the early starvation response rather than the depletion of metabolites in later stage.

## Discussion

The redox ratio, NAD^+^/NADH, is an important biological signature. Before the invention of a cycling assay [19], the ratio of free NAD^+^ and NADH concentrations was measured indirectly by quantifying the concentrations of products and substrates of dehydrogenase reactions that are known to be at equilibrium. This method was first developed in the Krebs lab using these enzyme systems [27]: lactate dehydrogenase (LDH, E.C. 1.1.1.27) in cytosol, β–hydoxybutyrate dehydrogenase in mitochondrial cristae (EC 1.1.1.30) and malate dehydrogenase (MDH, E.C. 1.1.1.37) in the mitochondrial matrix. However, these assays not only assume the reactions are at equilibrium, but also assume the *K_m_*’s of the aforementioned dehydrogenases remain constant. (It should be pointed out that in their original paper, what was defined as *K_m_* is in fact dissociation constant, however). When comparing among different genetic backgrounds, the assumption of a constant *K_m_* does not always hold true. The association between genetic variation and catalytic efficiency at LDH, for example, is well documented and thought to be an important natural adaptation [33], [34], [35]. Very recently, Sun *et al*. showed that the equilibrium assumption is in fact hardly satisfied [36] for LDH. Hence, the [NAD^+^]/[NADH] can only be calculated from [lactate]/[pyruvate] when the reaction is at or close to equilibrium [36]. All these problems are avoided by direct measurement of the amount of pyridine nucleotide and subsequent calculation of the redox ratio, which does not rely on a constant *K_m_* nor reaction equilibrium. Direct NAD(P)x measurement should therefore provide improved resolution in comparisons among genotypes, genetic backgrounds or different metabolic states.

Direct measures of the quantity or concentration of pyridine nucleotides can be achieved in many ways but enzymatic recycling assays offer unique advantages. As NAD(P) recycles between the redox indicator dye reaction and the dehydrogenase reaction, each molecule can lead to the production of many molecules of reduced dye. The signal is therefore vastly amplified and high sensitivity can be achieved. Furthermore, an enzymatic assay is highly versatile and specific. By changing the dehydrogenase reaction this assay can be adopted for detecting different redox coenzymes and in this report both NADx and NADPx assay are shown. By replacing PES/MTT with PMS/resazurin, it can be turned into a sensitive fluorescence assay as well. Because the reaction depends on dehydrogenase, specificity can be granted by carefully selecting dehydrogenase specific to only NAD^+^ or NADP^+^ (for this reason, enzymes such as mammalian glutamate dehydrogenase EC 1.4.1.2 should be avoided as it is capable of using both NAD^+^ and NADP^+^ as substrate).

In rats, Williamson *et al*. [27] reported that during a shift from well-fed to starved, the free concentration ratio [NAD^+^]/[NADH] changes as follows: from 725 to 528 in cytosol and from 8 to 5 in mitochondria. They also showed that the ratio of total NAD^+^/NADH was 7.2 in cytoplasm and 2.2 in mitochondria hence most of NADH are in mitochondria and protein bound [27]. The result regarding the total amounts of pyridine nucleotides was obtained using a method contributed by Glock and McLean [37], in which in order to measure total amount of pyridine nucleotides, sample was divided in two parts and extracted separately. One extraction is made in acid for assaying NAD^+^ and the other in alkaloid for NADH assay. Using the enzyme cycling method, our finding of total NAD^+^/NADH being around 8 and halved with starvation agrees with these findings. NADPx predominantly exists in reduced form; we found the ratio of oxidized over reduced form to be around 0.2 for well-fed and decreased with starvation.

The concentrations of PMS and resazurin in this study are carefully chosen based on the study of reaction mechanism by Candeias *et al*. [38]. It was shown in that report that when the concentration of PMS exceeds 1000 µM, it can form secondary products. Note that the low concentration of dye will certainly limit the upper detection range.

This assay depends on the pH-dependent instability of pyridine nucleotides to distinguish between NAD(P)^+^ from NAD(P)H. In contrast to what has been reported before, we found 30 min of 65°C heat treatment alone cannot fully degrade NAD^+^ in fruit fly whole body homogenate (Table 1). As most of the NADx assays are optimized and tested for cell lines and mammalian tissue, we suspect this to be due to some unknown insect-specific metabolites which are capable of blocking NAD^+^ degradation by heat in neutral pH. It has also been suggested that two extractions be made from the same biological sample, one in acid and the other in alkali, and NAD^+^ and NADH can be assayed separately [19], [21], [26]. However, it has long been known as well that even high concentrations of OH^−^ or H^+^ cannot destroy the activity of pyridine nucleotide-consuming enzymes [26]. Considering these factors, we opt to prepare a common protein free homogenate and treat with OH^−^ or H^+^ after rather than before extraction.

It is important to minimize enzymatic degradation and to effectively dissociate protein bound NADH when aiming to measure its total amount. Both of these can be accomplished using the phenol chloroform extraction invented by Chomczynski and Sacchi [28]. After extraction, hydrophobic molecules, including proteins, are removed in chloroform phase while hydrophilic molecules remain in aqueous phase. Residual phenol chloroform carry-over did not seem to have a strong negative impact on the assay at least in the enzyme concentration reported in this study as enzyme has to be provided in excess amount.

Our approach, however, limits the choice of homogenization buffer. Carbonate based buffer [23] cannot be used as homogenization buffer as it will react with high concentration of H^+^. PO_4_^3−^ is known to speed up the degradation of pyridine nucleotides [24] and therefore should also be avoided. The additional nicotinamide in the buffer is for blocking NAD(P) to nicotinamide enzymatic degradation following an early report [23].

Adding a coupling reaction consuming acetaldehyde improves the ADH based NADx assay. The oxidation of ethanol catalysis by ADH not only has a positive ΔG^’0^, but when acetaldehyde is not removed the oxidation product of acetaldehyde has also been shown to interfere with ADH [39]. We show that using the Wolff–Kishner reduction as the coupling reaction to remove acetaldehyde improves the assay linearity especially at high concentrations. This additional coupled reaction increases *V_mean_* which leads to better sensitivity. However, it still does not improve the ADH reaction to a level comparable to that of G6PDH. The decay of velocity is still apparent after about 300 s. The standard curve is less linear (0.980 v.s. 0.995) and has a slightly smaller slope (8.11 v.s. 9.31) than NADPx assay using G6PDH (Fig. 2a, 2c, 2d and 2e). As shown above (Fig. 3b), one cannot further increase the concentration of hydrazine without ill effects. Acetaldehyde dehydrogenase (ALDH) coupling reaction may be a promising alternative but care must be taken to make sure it is free of bound NADH.

In this study, we adopted the enzymatic recycling assay of pyridine nucleotides to measure the redox ratio in fruit fly *Drosophila melanogaster* whole body samples. We improved the common protocol to address a special problem in fruit fly homogenate. We improved the linearity of ADH based NADx assay by adding a coupling reaction removing acetaldehyde. The protocol is shown to be suitable for assaying NADPx as well. We also suggested a way to effectively reduce degradation of pyridine nucleotides as well as to facilitate the releasing of pyridine nucleotides that are protein bound. As a proof of principle, we were able to use this assay to detect the reduction of redox ratio in fly tissues during short starvation. Given the growing interest in the role of redox balance in fitness, disease and aging, the improvements and ease of use promise to make this assay a useful tool in future studies.

## Supporting Information

Table S1 **List of abbreviations.**
(DOC)

Table S2 **List of Reagents.**
(DOC)

Materials and Methods S1 **Supplementary methods.**
(DOC)
