# Supplementary material for: A Hydrazine Coupled Cycling Assay Validates the Decrease in Redox Ratio under Starvation in Drosophila
Source: PLoS One. 2012 Oct 17;7(10):e47584. doi: 10.1371/journal.pone.0047584 (PMC3474733; doi:10.1371/journal.pone.0047584)
Supplement: Table S1 — List of abbreviations. (DOC) [file pone.0047584.s001.doc]

Table S1: List of abbreviations, in the order as they appear in the text:

| Full name | Abbreviation |  |
| --- | --- | --- |
| β-nicotinamide adenine dinucleotide, oxidized | NAD^+^ |  |
| β-nicotinamide adenine dinucleotide, reduced | NADH |  |
| β-nicotinamide adenine dinucleotide phosphate, oxidized | NADP^+^ |  |
| β-nicotinamide adenine dinucleotide phosphate, reduced | NADPH |  |
| alcohol dehydrogenase | ADH | (E.C. 1.1.1.1) |
| high-performance liquid chromatography | HPLC |  |
| Nuclear magnetic resonance | NMR |  |
| mass spectrometry | MS |  |
| 3-(4,5-Dimethylthiazol-2-yl)-2,5-diphenyltetrazolium bromide | MTT |  |
| phenazine ethosulfate | PES |  |
| N,N-bis(2-hydroxyethyl)glycine | BICINE |  |
| ethylenediaminetetraacetic acid | EDTA |  |
| phenazine methosulfate | PMS |  |
| glucose 6-phosphate | G6P |  |
| glucose 6-phosphate dehydrogenase | G6PDH | (E.C. 1.1.1.49) |
| glycerol phosphate oxidase | GPO | (E.C. 1.1.3.21) |
| acetaldehyde dehydrogenase | ALDH | (E.C. 1.2.1.10) |
| lactate dehydrogenase | LDH | (E.C. 1.1.1.27) |
| malate dehydrogenase | MDH | (E.C. 1.1.1.37) |
