# Supplementary material for: A Hydrazine Coupled Cycling Assay Validates the Decrease in Redox Ratio under Starvation in Drosophila
Source: PLoS One. 2012 Oct 17;7(10):e47584. doi: 10.1371/journal.pone.0047584 (PMC3474733; doi:10.1371/journal.pone.0047584)
Supplement: Table S2 — List of Reagents. (DOC) [file pone.0047584.s002.doc]

Table S2: List of reagents, in the order as they appear in the text:

| Name | Supplier | Catalog Number |
| --- | --- | --- |
| nicotinamide | Sigma-Aldrich | N0636 |
| N,N-Bis(2-hydroxyethyl)glycine | Sigma-Aldrich | B3876 * |
| phenazine ethosulfate | Sigma-Aldrich | A3263 |
| 3-(4,5-Dimethylthiazol-2-yl)-2,5-diphenyltetrazolium bromide | Sigma-Aldrich | M2128 |
| phenazine methosulfate | Sigma-Aldrich | P9625 |
| resazurin | Sigma-Aldrich | R7017 |
| β-nicotinamide adenine dinucleotide hydrate | Sigma-Aldrich | N7004 |
| β-nicotinamide adenine dinucleotide phosphate hydrate | Sigma-Aldrich | N5755 |
| β-nicotinamide adenine dinucleotide, reduced | Sigma-Aldrich | N6005 |
| β-nicotinamide adenine dinucleotide phosphate, reduced | Sigma-Aldrich | N6505 |
| hydrazine water solution | Sigma-Aldrich | 309400 |
| alcohol dehydrogenase | Sigma-Aldrich | A3263 ** |
| glucose 6-phosphate dehydrogenase | Sigma-Aldrich | G7877/G6378 *** |
| glucose 6-phosphate | Sigma-Aldrich | G7250 |
| phenol:chloroform:isoamyl alcohol (25:24:1) | Amresco | K169 |
| chloroform:isoamyl alcohol (24:1) | Amresco | X205 |
| amyloglucosidase | Roche Diagnostics | 11202332001 |
| Infinity ^TM^ Triglycerides reagent | Thermo Scientific | TR22421 |
| Infinity ^TM^ Glucose reagent | Thermo Scientific | TR15421 |
| Pierce BCA Protein Assay Reagent | Thermo Scientific | 23225 |
| *: Among several alternatives, this particular product requires very little adjustment to reach neutral | | |
| pH. | | |
| **: It is advisable to use Sigma-Aldrich product Cat. #A3263 as it has very low amount of NADH | | |
| remaining bound to the enzyme according to the manufacturer. | | |
| ***: Many other G6PDH products have to be avoided as they can react with either NADP^+^ or NAD^+^ | | |
| as cofactor (such as Sigma-Aldrich #G7877). | | |
