# Supplementary material for: A Hydrazine Coupled Cycling Assay Validates the Decrease in Redox Ratio under Starvation in Drosophila
Source: PLoS One. 2012 Oct 17;7(10):e47584. doi: 10.1371/journal.pone.0047584 (PMC3474733; doi:10.1371/journal.pone.0047584)
Supplement: Materials and Methods S1 — Supplementary methods. (DOC) [file pone.0047584.s003.doc]

### **Supplementary method:**

*Estimating redox ratio (NAD^+^/NADH) and testing the difference of redox ratio across groups.*

With both NAD^+^ and NADH having been measured from *each* biological sample (*y_i_* for NAD^+^ and *x_i_* for NADH in the *i*th sample), the least square estimate of NAD^+^/NADH redox ratio can be expressed as the regression coefficient term, *b*, estimated from the following constrained linear regression model. It is a constrained model because of the lack of the intercept term:

$\begin{matrix} y=b\cdot x \\ \text{which is equivalent to:} \\ b=\frac{y}{x}\text{ } \end{matrix}$ Equation 1.

Where *y* and *x* are the vectors of *y_i_* and *x_i_*.

Testing the treatment effect on the redox ratio between groups thus becomes a test of the difference of regression coefficients, *b*, of different groups. In the following model:

$\begin{matrix} y=b_{1}\cdot x+b_{2}\cdot T\cdot x \\ \text{where }T\text{ is a categorical variable:} \\ \left\{\begin{matrix} T=0\text{ ,for group 1} \\ T=1\text{ ,for group 2} \end{matrix}\right. \end{matrix}$ Equation 2.

When *T* = 0, *b*_1_ is the redox ratio for group 1; when *T* = 1, *b_1_+b_2_* is the redox ratio for group 2. When *b_2_* is significant, the regression coefficients for group 1 and group 2, hence the redox ratio in the two groups, are significantly different. Based on this principle, R program scripts were written for this study. Source code is available upon request.

Alternatively, one can calculate redox ratio for each sample:

$R_{i}=\frac{\overline{y}_{i}}{\overline{x}_{i}}\text{ }$ Equation 3.

where $\overline{y}_{i}$ and $\overline{x}_{i}$ are the mean concentration of NAD^+^ and NADH of the *i*th sample, assuming each sample has replicated measures. The treatment effect on redox ratio can be examined by *t*-test or analysis of variance (ANOVA). However, being a ratio, *R_i_* must be first properly transformed, e.g. by Box-Cox transformation, in order maintain the normality assumption of *t*-test and ANOVA. It must be pointed out that this approach may underestimate error terms because the measurement errors are ignored as the redox ratio for each sample is calculated from the means (equation 3). For this reason it is not used in this study.
